# Elevated circulating levels of an incretin hormone, glucagon-like peptide-1, are associated with metabolic components in high-risk patients with cardiovascular disease

**DOI:** 10.1186/1475-2840-9-17

**Published:** 2010-05-14

**Authors:** Minako Yamaoka-Tojo, Taiki Tojo, Naonobu Takahira, Atsuhiko Matsunaga, Naoyoshi Aoyama, Takashi Masuda, Tohru Izumi

**Affiliations:** 1 Department of Rehabilitation, Kitasato University School of Allied Health Sciences, 1-15-1 Kitasato, Minami-ku, Sagamihara, 252-0373, Japan; 2 Department of Cardioangiology, Kitasato University School of Medicine, 1-15-1 Kitasato, Minami-ku, Sagamihara, 252-0374, Japan

## Abstract

**Background:**

Glucagon-like peptide-1 (GLP-1) is an incretin hormone that has a wide range of effects on glucose metabolism and cardiovascular function (e.g., improving insulin sensitivity, reduction in appetite, modulation of heart rate, blood pressure and myocardial contractility). Metabolic syndrome (MetS) is associated with an increased risk of developing atherosclerotic cardiovascular diseases. Novel glycemic control drugs, the dipeptidyl-peptidase-4 (DPP-4) inhibitors, work by inhibiting the inactivation of incretin hormones, GLP-1 and glucose-dependent insulinotropic polypeptide (GIP). In spite of good effects of these drugs in diabetic patients, circulating levels of incretins and their role in MetS are largely unknown.

**Methods:**

To examine relationships between incretin hormones and MetS risk factors, we measured circulating levels of incretins in obese high-risk patients for cardiovascular disease. Fasting serum GLP-1 and GIP levels were measured by ELISA. We performed a cross-sectional analysis of metabolic variables in the fasting state in two subject groups: with MetS (n = 60) and pre-MetS (n = 37).

**Results:**

Fasting levels of Serum GLP -1 in the peripheral circulation were significantly increased correlated with the accumulation of MetS risk factors components (*r *= 0. 470, *P *< 0.001). There was a significant interaction between circulating GLP-1 and GIP, serum high-density lipoprotein cholesterol, triglyceride, and serum uric acid concentrations but not waist circumference, fasting glucose, HbA1c, or presence of diabetes.

**Conclusion:**

Circulating levels of GLP-1 in relation to the accumulation in MetS factors suggested that MetS patients with elevated levels of GLP-1 are high-risk patients for cardiovascular disease, independent with the presence of diabetes.

## Background

The metabolic syndrome (MetS) is a major public health problem [1] and a multiple risk factor for cardiovascular disease [2,3]. It consists of atherogenic dyslipidemia (elevated triglycerides and low high-density lipoprotein [HDL]), and elevations of blood pressure and glucose, and abdominal obesity with pro-thrombotic and proinflammatory states [1]. MetS is associated with a 5-fold higher risk of developing type 2 diabetes and 2.6-to 3-fold high risk of cardiovascular disease [4,5]. The pathophysiology of MetS is not well defined, and several investigators have sought to identify a unifying factor that could explain all the components of the syndrome. In addition to insulin resistance/hyperinsulinemia, investigators have found several biomarkers to be associated with MetS including leptin [4], catecholamine [6], brain natriuretic peptide (BNP) [7], oxidized low-density lipoprotein cholesterol (LDL)[8], uric acid [9], C-reactive protein (CRP) [4], plasminogen activator inhibitor-1 [4], aldosterone [4], cyctatin C [10], and carboxy-terminal prevasopress in (copeptin)[11], highlighting diverse pathophysiological perturbations in MetS [11].

Glucagon-like peptide-1 (GLP-1) is a hormone derived from the prepro-glucagon molecule and is secreted by intestinal L cells [12]. It is the most potent stimulator of glucose-induced insulin secretion and also suppresses *in vivo *acid secretion by gastric glands. Intracerebroventricular GLP-1 is powerfully inhibited in fasting rats [13]. In addition, injection of a specific antagonist of GLP-1 blocked the inhibitory effect of GLP-1 on food intake. GLP-1 receptor is expressed in the central nervous system or in the stomach except β cells of the pancreas, and its every function such as inhibition of insulin secretion, appetite suppression and gastric motor inhibition has a hypoglycemic effect. Thus, the development of GLP-1-releated study as a diabetic drug (e.g., GLP-1 analogs and the dipeptidyl-peptidase-4 [DPP-4] inhibitors) is progressing.

We therefore hypothesized that circulating GLP-1 would be associated with insulin resistance/hyperinsulinemia and MetS. To examine relationships between incretin hormones and MetS components, we measured circulating levels of incretins, GLP-1 and gastric inhibitory polypeptide (GIP), in high-risk patients for cardiovascular disease.

## Methods

### Subjects

The study included 97 Japanese high-risk outpatients for cardiovascular disease with abdominal obesity (40% female) in the Obesity/Metabolic Syndrome Clinic, Department of Cardioangiology, Kitasato University Hospital. The only exclusionary criterion at enrolment was the treatment with antidiabetic drugs including insulin and oral agents. All subjects gave informed consent before participating in this study, and the ethics committee of the Kitasato University Hospital approved the study design. Body mass index (BMI) was calculated as weight divided by height squared. Systolic and diastolic blood pressure was measured after a rest for at least 15 minutes with a sphygmomanometer in sitting position. Homeostasis model assessment (HOMA-IR) was used as a measure of insulin resistance and was calculated as fasting plasma insulin (μU/mL) × glucose (mg/dL)/405 [14].

Metabolic scores were calculated using MetS components according to the Japanese MetS criteria [15]. The score consisted of four independent components as abdominal obesity, defined as a waist circumference ≥85 cm in men or ≥90 cm in women, hypertriglyceridemia and/or low HDL-cholesterolemia, hypertension, and elevated fasting glucose. The diagnosis of hypertension was established based on blood pressure levels measured at the study visit (≥130/85 mmHg) or a prior diagnosis of hypertension and current treatment with antihypertensive medications. Diabetes/impaired fasting glucose (IFG) was considered present if the subject had history of diabetes or had a fasting glucose level of 110 mg/dL or greater.

### Measurement of clinical biomarkers

Blood samples were collected by venipuncture after an overnight fast. Serum was centrifuged (1500 g for 15 min at 4°C) and stored at 4°C until measurement within couple days for biochemical markers, such as triglyceride, LDL cholesterol, HDL cholesterol, insulin, plasma glucose, glycated haemoglobin (HbA1c), uric acid, CRP, and BNP were measured enzymatically in the clinical laboratory of Kitasato University Hospital.

### Measurement of incretin hormones

Circulating levels of human GLP-1 and GIP were determined by enzyme-linked immunosorbent assay (ELISA) using the GLP-1 (7-36) amide/prepro-glucagon (98-127) amide enzyme immuno assay EIA kit (Phoenix Pharmaceuticals, Inc., Burlingame, CA) and the human GIP assay kit (Immuno-Biological Laboratories Co., Ltd., Takasaki, Japan), respectively.

### Statistical analysis

Continuous data were summarized as either mean ± SD or median and quartiles, and categorical data were expressed as percentages. Data were compared by unpaired *t*-test or Mann-Whitney *U*-tests where appropriate. Differences in proportions of variables were determined by chi-squared analysis. To evaluate the correlations between GLP-1 and selected variables, we calculated Spearman correlation coefficients between circulating levels of fasting GLP-1 and the following variables: 1)conventional risk factors for cardiovascular disease (LDL cholesterol, HDL cholesterol, triglyceride, HbA1c, presence of hypertension, and current smoking), and history of coronary artery disease; 2) measures of adiposity and insulin resistance (BMI, waist circumference, fasting blood glucose, insulin level, and HOMA-IR); and 3) metabolic risk scores (abdominal obesity, hypertension, high triglyceride and/or low HDL cholesterol, and glucose intolerance or diabetes) and MetS-related co-morbidity (hyper uric academia, fatty liver disease, chronic kidney disease, and sleep apnea syndrome). Participants having low metabolic scores (0, 1, or 2) was termed the pre-MetS whereas patients having high metabolic scores (3 or 4) were defined as MetS.

To evaluate the association of GLP-1 with MetS, we constructed multivariable logistic regression models to assess whether circulating GLP-1 was independently associated with MetS. We calculated the odds ratio for the presence of MetS in quartiles of GLP-1 with participants in the lowest quartile of GLP-1 considered the referent group. Adjustments were performed for age and sex; and age, sex, and beta-blocker use. Two-sided *P*-values < 0.05 were considered significant.

## Results

### Clinical characteristics and metabolic variables

Sixty patients of MetS and 37 patients of pre-MetS with atherosclerosis-prone conditions participated in the study. The patients with MetS were older than pre-MetS patients but the proportion of women was not different between the groups (Table 1). In MetS patients, BMI was greater than in pre-MetS patients (average BMI 32.1 *vs. *29.0, *P *= 0.015). Thirty-seven of patients (62%) with MetS were hypertension and used one or more antihypertensive drugs. A half of the patients with MetS were treated with calcium-channel blockers. Twelve patients with MetS and three patients with pre-MetS were diagnosed IFG or type 2 diabetes. None of patients received antidiabetic drugs including insulin. Fasting plasma glucose levels were higher in patients with pre-MetS than with MetS (*P *= 0.025). Predictably, HDL cholesterol was lower in the patients with MetS compared with pre-MetS (*P *= 0.001). Thirty-eight of the patients with MetS were diagnosed dyslipidemia and 45% of the patients received statins whereas only 19% of the patients with pre-MetS used statins (*P *= 0.016). The proportion of participants who have previous and/or current coronary artery disease was significantly greater in patients with MetS than those in pre-MetS (*P *= 0.027). However, circulating levels of BNP, a useful biomarker for heart failure, did not differ between the 2 groups.

**Table 1:** Participant characteristics (n = 97).

	MetS (n = 60)	Pre-MetS (n = 37)	*P*-value
Age (year)	52.8 ± 14.2	50.3 ± 16.6	0.494
Sex, female (%)	22 (36.7%)	17 (45.9%)	0.333
BMI (kg/m^2^)	32.1 ± 6.5	29.0 ± 4.9	0.015
Waist circumference (cm)	103 ± 15	98 ± 11	0.093
Plasma glucose (mg/dL)	114 ± 28	130 ± 41	0.025
Plasma insulin (IU/mL)	33.0 ± 36.4	33.4 ± 37.5	0.961
HOMA-IR	4.8 ± 6.6	4.7 ± 7.0	0.944
LDL cholesterol (mg/dL)	129 ± 33	121 ± 36	0.366
HDL cholesterol (mg/dL)	50.5 ± 11.9	61.1 ± 12.4	0.001
Triglyceride (mg/dL)	235 ± 224	151 ± 128	0.200
HbA1c (%)	5.7 ± 0.9	5.8 ± 1.0	0.605
CRP (μg/dL)	254 ± 575	156 ± 226	0.323
Smoking, n (%)	14 (23.3%)	11 (29.7%)	0.484
Hypertension, n (%)	37 (61.7%)	20 (54.1%)	0.623
Diabetes/IFG, n (%)	21 (35.0%)	14 (37.8%)	0.658
Dyslipidemia, n (%)	38 (63.3%)	18 (48.6%)	0.236
Coronary artery disease, n (%)	13 (21.7%)	2 (5.4%)	0.027
Hyper uric acidemia	14 (23.3%)	10 (27.0%)	0.596
Fatty liver disease	10 (16.7%)	11 (29.7%)	0.102
Chronic kidney disease	5 (8.3%)	2 (5.4%)	0.627
OSAS, n (%)	4 (6.7%)	1 (2.7%)	0.416
Statin use, n (%)	27 (45.0%)	7 (18.9%)	0.016
Diuretic use, n (%)	12 (20.0%)	6 (16.2%)	0.658
β-blockers, n (%)	21 (35.0%)	13 (35.1%)	0.899
RAAS inhibitors, n (%)	10 (16.7%)	6 (16.2%)	0.985
Ca-channel blockers, n (%)	31 (51.7%)	14 (37.8%)	0.326
Aspirin, n (%)	21 (35.0%)	7 (18.9%)	0.117
Metabolic score (0, 1, 2, 3, and 4)	3.3 ± 0.5	1.5 ± 0.7	<0.001

Continuous variables are presented as mean ± SD whereas categorical variables are presented as counts and percentages. BMI, body mass index; HOMA-IR, homeostatis model assessment; LDL, low density lipoprotein; HDL, high density lipoprotein; HbA1c, glycated haemoglobin; IFG, impaired fasting glucose; OSAS, obstructive sleep apnea syndrome; RAAS, rennin-angiotensin-aldosterone system; Ca-channel, calcium channel.

### Circulating levels of GLP-1 are increased in MetS

**Figure 1:**
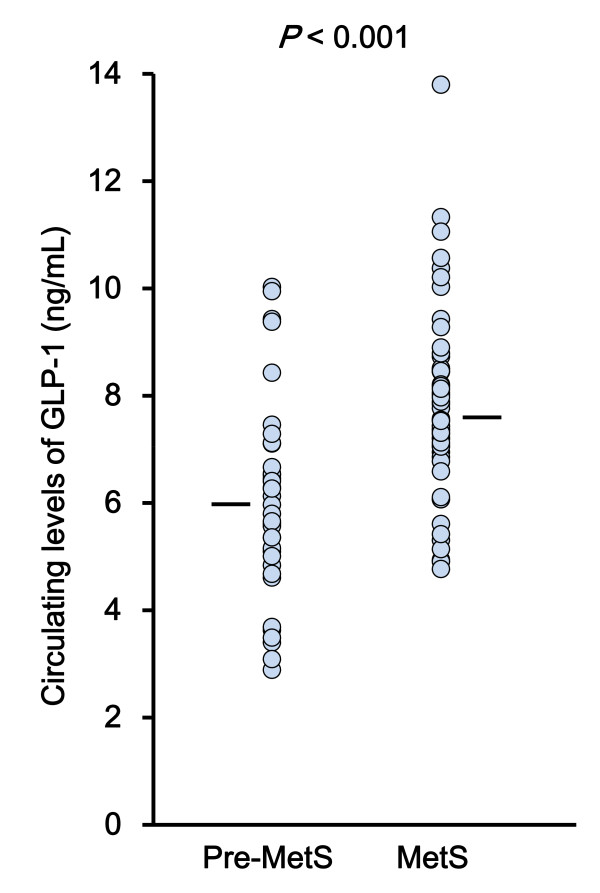
**Circulating levels of glucagon-like peptide-1 (GLP-1) in patients with metabolic syndrome (MetS) and pre-MetS conditions**. Scatter plot showing the GLP-1 (ng/mL) in patients with pre-MetS conditions (n = 37) and MetS (n = 60). The bars indicate the mean values.

**Figure 2:**
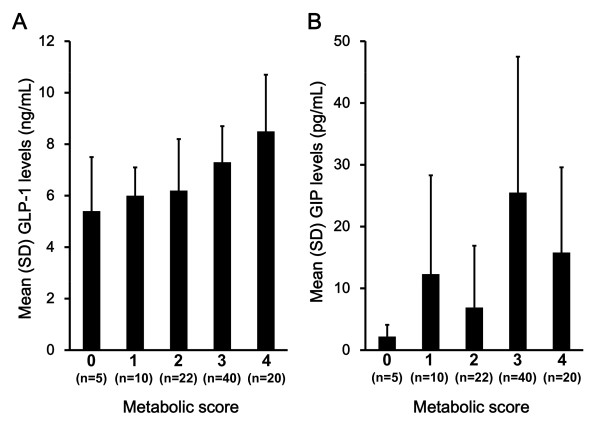
**Association between serum levels of incretins, glucagon-like peptide-1 (GLP-1) and gastric inhibitory polypeptide (GIP), and metabolic syndrome components in patients with atherosclerosis-prone conditions**. Circulating levels of GLP-1 (A) and GIP (B) were measured in high-risk patients with atherosclerosis. Metabolic score is the number of metabolic syndrome components; abdominal obesity, hypertension, dyslipidemia (high triglyceride and/or low high density lipoprotein cholesterol), and hyperglicemia (impaired glucose tolerance or diabetes). Progressive increase in GLP-1 level as a function of the number of MetS components (*P *< 0.001).

As shown in Figure 1, the serum GLP-1 concentration was on average 28% higher in MetS patients (7.7 ± 1.9 ng/mL)compared to pre-MetS subjects (6.0 ± 1.6 ng/mL, *P *< 0.001). This difference remained significant after controlling for age and sex (*P *= 0.010). When the data from the patients with MetS and pre-MetS were combined, GLP-1 levels were similar in men (7.0 ± 2.0 ng/mL) and women (7.2 ± 2.0 ng/mL) (*P *= 0.709). We next compared patient groups on the basis of the number of MetS criteria that were concomitantly present (Figure 2). Serum GLP-1 levels elevated with increasing number of MetS components (*P *< 0.001; Figure 2A). Among MetS components, the presence of dyslipidemia (high triglyceride and/or low HDL cholesterol) was significantly related to circulating levels of GLP-1. On the other hand, there was no correlation between the accumulation of MetS components and serum levels of GIP (Figure 2B).

### Association of serum GLP-1 and GIP with clinical variables

Circulating GLP-1 positively correlated with fasting serum triglycerides, uric acid, and CRP, and inversely correlated with HDL cholesterol (Table 2). Above all, serum GLP-1 was powerfully associated with a useful biomarker for systemic inflammation, serum CRP(*r *= 0.550, *P *= 0.026). In addition, circulating levels of GIP were significantly associated with GLP-1 (*r *= 0.383, *P *< 0.001). Interestingly, serum GIP was positively correlated with serum levels of LDL cholesterol and hyper uric acidemia with or without medications. However, no significant correlation was observed between serum GIP and the stating or allopurinol usage. In addition, there was no correlation between serum GIP and MetS scores. Although each variable has a large dispersion, the serum GIP concentration was on average three times higher in MetS patients (19.0 ± 45.7 pg/mL) compared to pre-MetS subjects (6.5 ± 10.2 pg/mL) (*P *= 0.034).

**Table 2:** Spearman correlations between GLP-1, GIP, and selected variables in patients with MetS and pre-MetS conditions (n = 97).

	*GLP-1 r*	*P-*value	*GIP r*	*P-*value
Age (years)	0.004	0.145	0.009	0.938
Sex	0.202	0.046	-0.063	0.603
Waist	0.078	0.464	0.274	0.310
BMI	0.089	0.747	0.486	0.056
LDL cholesterol (mg/dL)	0.204	0.093	0.357	0.003
HDL cholesterol (mg/dL)	-0.229	0.049	-0.009	0.941
Triglyceride (mg/dL)	0.243	0.023	0.041	0.730
Smoking	0.118	0.326	-0.133	0.274
CRP (μg/dL)	0.550	0.026	-0.047	0.694
Plasma glucose (mg/dL)	0.064	0.554	-0.388	0.140
Plasma insulin (IU/mL)	0.069	0.571	0.088	0.499
HOMA-IR	-0.116	0.284	0.013	0.920
Diabetes/IFG	0.049	0.652	-0.132	0.278
HbA1c	-0.303	0.212	-0.029	0.810
Hypertension	0.059	0.589	0.197	0.099
Dyslipidemia	0.031	0.773	0.011	0.925
Coronary artery disease	0.093	0.391	-0.094	0.439
Hyper uric acidemia	0.133	0.219	0.276	0.019
Serum uric acid (mg/dL)	0.245	0.025	-0.023	0.849
Fatty liver disease	-0.054	0.618	-0.115	0.340
Chronic kidney disease	0.029	0.793	-0.046	0.703
OSAS	0.178	0.099	-0.014	0.908
Metabolic score	0.470	<0.001	0.157	0.189
GIP (pg/mL)	0.383	<0.001		

GLP-1, glucagon-like peptide-1; GIP, gastric inhibitory polypeptide; *r*, Spearman correlation coefficients; BMI, body mass index; LDL, low density lipoprotein; HDL, high density lipoprotein; CRP, C-reactive protein; HOMA-IR, homeostatis model assessment; IFG, impaired fasting glucose; HbA1c, glycated haemoglobin; OSAS, obstructive sleep apnea syndrome.

### Association of serum GLP-1 with the presence of MetS

In multivariable logistic regression analyses that adjusted for age and sex, circulating GLP-1 levels in the forth quartile were significantly associated with higher odds ratios (OR) of having MetS (Table 3). This association remained significant after additional adjustment for statin use, history of coronary artery disease, and diuretic use, with OR of 8.04 (*P *= 0.015) for the fourth GLP-1 quartile.

**Table 3:** Association of serum GLP-1 with the presence of MetS: logistic regression analyses.

	OR (95% CI)			
	
	*P *value			
	
	First quartile	Second quartile	Third quartile	Fourth quartile
GLP-1 (ng/mL)	<5.7	5.7-7.2	7.2-8.2	>8.2
Unadjusted	1	0.68 (0.14-3.35) 0.643	3.67 (0.87-15.4) 0.076	5.16 (1.23-21.56) 0.025
Age adjusted	1	0.746 (0.15-3.86) 0.746	3.75 (0.89-15.9) 0.072	5.56 (1.30-23.9) 0.021
Fully adjusted	1	0.777 (0.13-4.56) 0.780	3.57 (0.72-17.7) 0.119	8.036 (1.50-42.9) 0.015

The fully adjusted model includes age, sex and body mass index. OR, odds ratios; CI, confidence interval; GLP-1, glucagon-like peptide-1.

## Discussion

The present study, to the best of our knowledge, is the first to report that circulating fasting levels of GLP-1 is associated with the accumulation in MetS components. Participants with MetS had higher mean GLP-1 levels than those without MetS and participants in the fourth quartiles for serum GLP-1 had markedly higher odds of MetS compared with those in the bottom quartile (Table 3). In addition, serum GLP-1 levels were increased in participants who had greater clustering of MetS components (Figure 2A). These associations were independent of adiposity or the presence of diabetes/IFG. Furthermore, serum GLP-1 was positively correlated with serum CRP. These data suggested that MetS patients with elevated levels of GLP -1 are high-risk patients for cardiovascular disease, independent with the presence of diabetes. Gastrointestinal hormones (such as GLP-1 or GIP) that come under incretin, are secreted by intestine in response to nutrient ingestion [16]. Originally, it has been thought that they are gastrointestinal factors involved in post-ingestion augmented secretion of insulin by β cell of the pancreas [17]. According to previous literature, GLP-1 and GIP are both secreted within minutes ingestion and facilitate the rapid disposal of ingested nutrients [18]. GLP-1 promotes satiety and sustained GLP-1 receptor activation is associated with weight loss in both preclinical and clinical studies [19]. As shown in Figure 2A, circulating levels of GLP-1 were correlated with MetS score in patients with MetS. On the other hand, there was no significant correlation between circulating GIP and the MetS score (Table 2). According to a previous report, circulating GIP was not elevated in diabetic patients [20]. One of the reasons why there was no correlation between circulating GIP and MetS component may be due to large dispersion of circulating GIP levels in these patients. Unfortunately, we don't have enough data to determine a role on elevated levels of circulating GIP in patients with MetS. In a previous report about GLP-1 or GIP infusion to human, GLP-1 is a physical incretin and more powerful than GIP [16]. Therefore, we focused on circulating levels of GLP-1 in the present study. Earlier reports showed that GIP receptor is expressed in cells, such as β cells of the pancreas, adipocytes, or osteoblastic cells, and it plays essential roles in reserving inghested nutrients within the body in each cell. It has been reported that the control of the GIP signal can lead to improvement of MetS [21-24]. According to a previous study using mice with an inactivated GIP receptor, the duodenal hormone GIP directly links over-nutrition to obesity [22]. In a previous animal model, double incretin receptor knockout mice (Glp1r^-/- ^and Gipr^-/-^) fed a high-fat diet exhibited increased energy expenditure [24]. In recent studies, GLP-1 receptors are expressed in the pancreas, brain, heart, vasculature, lung, kidney, and gastrointestinal tract [17]. These data suggest that circulating levels of GLP -1 may affect to systemic metabolism in multiple organs including cardiovascular systems as a multifunctional hormone.

Recent studies have demonstrated that GLP-1 receptor agonists have wide-ranging cardiovascular actions, such as modulation of heart rate, blood pressure, vascular tone, and myocardial contractility [25]. Importantly, is appears that these agents may also have beneficial effects in the setting of cardiovascular disease. For example, GLP -1 has been found to exert cardioprotective actions in experimental models of dilated cardiomyopathy, hypertensive heart failure, and myocardial infarction. Moreover, preliminary clinical studies also indicate that GLP-1 infusion may improve cardiac contractile function in chronic heart failure patients with and without diabetes, and in myocardial infarction patients after successful angioplasty [26,27]. The precise mechanisms underlying the beneficial effects of GLP -1 in cardiac ischemia have yet to be established [25]. However, several experimental studies indicate that they occur independently of effects on glucose metabolism and may involve activation of cyclic guanosine monophosphate/cyclic adenosine monophosphate-dependent pathways and prosurvival kinases such as PI3K, Akt, glycogen synthase kinase-3β, p70s6 kinase, ERK1/2, and p38 MAPK [28-33].

Although further basic mechanistic research together with clinical investigations and metaanalyses are required, GLP-1-related drug compounds could exert beneficial effects on not only diabetes but also the cardiovascular system in patients with MetS. Several studies have shown that the magnitude of nutrient-stimulated insulin secretion is diminished in subjects with type 2 diabetes, promoting investigation as to whether incretin secretion and/or incretin action is diminished in diabetic subjects [34]. Plasma levels of GIP appear normal to increased in subjects with type 2 diabetes, whereas meal-stimulated plasma levels of GLP-1 are modestly but significantly diminished in patients with impaired glucose tolerance and in subjects with type 2 diabetes [20,35]. Based on the results of this clinical study, significant differences were found in fasting serum levels of GLP-1 between the MetS group and the pre-MetS group. On the other hand, no significant differences in GLP-1 concentration between the diabetes/IFG group and the control (normal fasting glucose) group (data not shown). The reason of the difference may come from the diabetes/IFG group including only early stage diabetes patients untreated with antidiabetic agents but not moderate to severe diabetes patients treated with oral antidiabetic agents and/or insulin.

Although the mechanism of elevated levels of GLP-1 in MetS is largely unknown, secretion of GLP-1 mostly depends upon the specific nutrient composition of the meal, and it has been reported that a particular caloric threshold or nutrient delivery rate must be reached in order to trigger significant secretion [36]. Generally, MetS patients tend to have a binge -eating disorder and it may be one of the causes of elevated levels of GLP-1 in patients with MetS. Even though the results of the study revealed no significant differences in fasting insulin and HOMA -IR between the MetS group and the pre-MetS, the levels of fasting glucose were significantly increased in the MetS group. Thus, these results suggest that the accumulation of MetS components induces the elevation of serum GLP-1 accompanied by increased levels of CRP (Table 1). Based on these observations, it is thus possible that high levels of GLP-1 may exhibit predictive information for atherosclerosis in patients with MetS.

In the present study, we found serum GLP-1 to be independently associated with several components of MetS including adiposity (BMI) and dyslipidemia (lower HDL cholesterol and/or high triglyceride levels) (Table 2). In our speculation, the association of GLP-1 with higher triglyceride levels may be secondary to increased hepatic synthesis of triglycerides under influence of glucocorticoids, glucagon, and obesity and/or MetS -induced sympathetic nervous system activation [11].

Interestingly, fasting levels of GLP-1 were significantly lower in patients after gastric bypass surgery compared with morbidly overweight controls [37,38]. Gastric bypass causes rapid resolution of insulin resistance and improved insulin secretion, perhaps mediated in part by increased GLP-1 [39], even before major weight loss has been achieved [40]. Although our study cannot demonstrate causation, it is important to note that fasting levels of GLP-1 might be linked to improve insulin resistance in obese patients. In this respect, circulating GLP-1 may be a novel biomarker for improving insulin sensitivity in high -risk patients for cardiovascular disease.

Concerning the glucose metabolism, excess glucagon secretion, abnormally accelerated gastric emptying during hyperglycemia, obesity, and increased food intake all contribute to hyperglycemia [41]. The progressive impairment of β cell function and increased insulin demand as tissue becomes insulin resistance are core pathophysiologic defects in the development of hyperglycemia in type 2 diabetes [42,43]. One of the major mechanisms of the genesis and progression of type 2 diabetes is progressive ectopic lipid deposition (e.g., in myocytes and hepatocytes, rather than in adipocytes), which induces insulin resistance, cell lipotoxicity, and diminished cell function, leading to metabolically inadequate insulin secretion [44]. Impaired release or action of GLP-1 increases excessive insulin secretion, and may play a role in the development and/or progression of type 2 diabetes in patients with MetS [45,46]. Furthermore, postprandial GLP-1 response is positively associated with changes in neuronal activity of brain areas implicated in satiety and food intake regulation in humans [47]. In a previous report, circulating levels of GLP-1 were decreased in patients with type2 diabetes [31].

**Figure 3:**
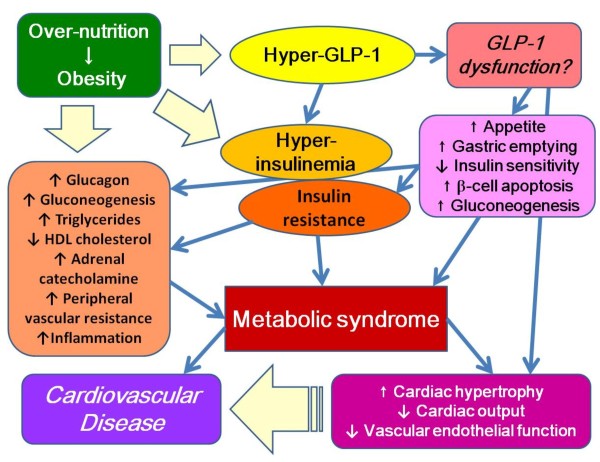
**Mechanism by which excessive circulating levels of GLP-1 may lead to insulin resistance and metabolic syndrome**. GLP-1, glucagon-like peptide-1.

In a recent study, a GLP-1 analog was effective on obese patients compared to a previous anti-obese drug, olristat. From these data, we speculate that elevating levels of GLP-1 in patients with MetS suggest the presence of systemic hyper GLP -1 concentration-induced "GLP-1 resistance" or "GLP-1 dysfunction" in those patients like as insulin resistance or leptin resistance (Figure 3). This new concept may shed further light upon the mechanisms involved in the GLP-1-inducing effect on the systemic metabolic disorder. In this point, treatment with GLP-1 mimetic agents, including DPP-4 inhibitors and GLP-1 analogs, may be a novel therapeutic strategy for patients with not only diabetes but also MetS and atherosclerosis-prone conditions.

## Conclusions

In summary, higher circulating levels of GLP-1 are associated with accumulations of MetS scores and systemic inflammation, independent of the presence of diabetes. Circulating GLP-1 may be a novel biomarker for high-risk patients with MetS, and further studies are warranted to assess its utility as a predictor of incident MetS and atherogenic conditions. Our findings may suggest a novel pathophysiological mechanism underlying over nutrition, elevated levels of circulating GLP-1, and metabolic syndrome.

## Competing interests

MY- T has served as a consultant to Bayer Inc. regarding the development of drugs for the treatment of atherosclerosis. The remaining authors declare that they have no competing interests.

## Authors' contributions

MY- T participated in the design of the study and performed the statistical analysis. TT conceived of the study, and participated in its design and coordination and helped to draft the manuscript. Other authors participated in enrolling patients in the study and discussion. All authors read and approved the final manuscript.
